# S100A12 is up-regulated in pulmonary tuberculosis and predicts the extent of alveolar infiltration on chest radiography: an observational study

**DOI:** 10.1038/srep31798

**Published:** 2016-08-19

**Authors:** Luis C. Berrocal-Almanza, Surabhi Goyal, Abid Hussain, Tilman E. Klassert, Dominik Driesch, Zarko Grozdanovic, Gadamm Sumanlatha, Niyaz Ahmed, Vijayalakshmi Valluri, Melanie L. Conrad, Nickel Dittrich, Ralf R. Schumann, Birgit Lala, Hortense Slevogt

**Affiliations:** 1 Institute for Microbiology and Hygiene, Charité-Universitätsmedizin Berlin, Berlin, Germany; 2 Septomics Research Center, Jena University Hospital, Jena, Germany; 3 Department of Immunology, Bhagwan Mahavir Medical Research Centre, Hyderabad, India; 4 BioControl Jena GmbH, Jena, Germany; 5 Department of Radiology, Campus Benjamin Franklin, Charité - Universitätsmedizin Berlin, Germany; 6 Pathogen Biology Laboratory, Department of Biotechnology and Bioinformatics, University of Hyderabad, Hyderabad, India; 7 Immunology and Molecular Biology, LEPRA Society- Blue Peter Research Centre, Hyderabad, India; 8 Department of Paediatric Radiology, Campus Virchow-Klinikum, Charité - Universitätsmedizin Berlin, Germany

## Abstract

Pulmonary tuberculosis (PTB) results in lung functional impairment and there are no surrogate markers to monitor the extent of lung involvement. We investigated the clinical significance of S100A12 and soluble receptor for advanced glycation end-products (sRAGE) for predicting the extent of lung involvement. We performed an observational study in India with 119 newly diagnosed, treatment naïve, sputum smear positive, HIV-negative PTB patients and 163 healthy controls. All patients were followed-up for six months. Sociodemographic variables and the serum levels of S100A12, sRAGE, esRAGE, HMGB-1, TNF-α, IFN-γ and CRP were measured. Lung involvement in PTB patients was assessed by chest radiography. Compared with healthy controls, PTB patients had increased serum concentrations of S100A12 while sRAGE was decreased. S100A12 was an independent predictor of disease occurrence (OR 1.873, 95%CI 1.212–2.891, p = 0.004). Under DOTS therapy, S100A12 decreased significantly after 4 months whereas CRP significantly decreased after 2 months (p < 0.0001). Importantly, although CRP was also an independent predictor of disease occurrence, only S100A12 was a significant predictor of lung alveolar infiltration (OR 2.60, 95%CI 1.35–5.00, p = 0.004). These results suggest that S100A12 has the potential to assess the extent of alveolar infiltration in PTB.

Tuberculosis (TB) is a global public health problem, particularly in low-income and middle-income countries which account for more than 80% of the cases^1^. Active TB most frequently affects the lungs and its pathology is often characterized by an exudative reaction progressing to caseous pneumonia^2^. In pulmonary TB (PTB) the extent of tissue pathology is associated with bacillary burden and impairment of lung function^2^,^3^. Importantly, the extent of pulmonary affliction during active disease is also associated with long term function impairment which often persists after successful completion of antibiotic treatment^4^,^5^. Current strategies to diagnose and follow active TB are based on microbiological, clinical and radiographic examinations^6^. However, the extent of pulmonary affliction at the time of diagnosis is rarely quantified, mainly due to the lack of surrogate markers and standardized evaluation systems. Thus, besides the urgent need for better markers to diagnose active PTB, the identification of surrogate markers for stratification of lung involvement might aid the formulation of step care plans to address the possibility of long-term lung impairment^7^.

There is increasing evidence from experimental *M. tuberculosis* infection in mice that the influx of neutrophils into the lungs is associated with necrotic lesions and increased pulmonary pathology^8^,^9^,^10^. Human S100A12 is predominantly expressed and secreted by activated neutrophil granulocytes^11^. In this regard, S100A12 is known to trigger a pro-inflammatory immune response by binding the multi-ligand receptor for advanced glycation end-products (RAGE) and activating transcription factors such as the nuclear factor (NF)-kB^11^,^12^,^13^,^14^. In addition to the membrane-bound full-length RAGE, two soluble RAGE forms exist; a soluble endogenous secreted form (esRAGE) produced by alternative splicing, and soluble cleaved RAGE (sRAGE) produced after the proteolytic cleavage of full-length membrane bound RAGE by metalloproteinases^12^,^13^,^15^,^16^,^17^,^18^. Notably, these soluble forms act as decoy receptors that counteract the proinflammatory processes triggered by the RAGE/ligand interaction^12^.

Serum levels of the RAGE ligands S100A12 and HMGB1 as well as the soluble RAGE forms sRAGE and esRAGE, have been shown to be differently regulated during chronic inflammatory diseases, and are proposed as correlates of disease severity and outcome^19^,^20^,^21^. The aim of the present study was to analyse the association between serum levels of sRAGE, esRAGE, S100A12 and HMGB1 and the extent of lung involvement assessed by Chest X-rays (CXR) in patients with smear positive PTB.

## Results

### Serum levels of S100A12 and sRAGE are differently regulated in PTB patients and healthy controls

(Table 1) portrays overall and analysis-specific characteristics of the 119 patients with PTB and the 163 healthy controls. The cases were younger than the controls (26 ± 11 vs 32 ± 10 years, p < 0.0001) and had a lower BMI (16 ± 2.6 vs 24 ± 4.5 kg/m2, p < 0.0001). There were no significant differences in gender between groups. The serum levels of sRAGE, esRAGE, S100A12, HMGB1 along with C-reactive protein (CRP) were assessed for association with PTB. After one initial comparison, different adjustments were done to the crude significance values according to the existing evidence regarding their relationship with PTB or with levels of sRAGE, esRAGE, S100A12, HMGB-1 and CRP, respectively^22^,^23^,^24^,^25^,^26^,^27^,^28^,^29^. After adjustments, a higher frequency of patients with cigarette smoking and alcohol consumption could be found when compared to controls (0.21 ± 0.83 vs 0.07 ± 0.28 pack-years) and (19% vs 2%), (p = 0.004 and < 0.0001) respectively. In addition, cases had lower systolic blood pressure (BP), (103 ± 15 vs 123 ± 17 mm/Hg, p < 0.0001). As shown in (Table 1), CRP was higher in cases than in controls; in contrast the sRAGE serum level was lower in cases than in controls, while the serum levels of S100A12 and TNF-α were higher in cases than in controls. There were no crude or adjusted differences in the levels of IFN-γ, HMGB-1 and esRAGE (Table 1). These data suggest that, TNF-α, S100A12, sRAGE and CRP are associated with PTB.

### S100A12 and CRP are independent predictors of PTB disease occurrence

In a next step, we used uni- and multivariable logistic regression analyses to evaluate whether the variables with significant adjusted differences could serve as independent predictors of disease occurrence. In the univariable models, serum levels of CRP, S100A12, sRAGE and TNF-α, as well as age, BMI, systolic BP, and alcohol consumption were predictors of disease occurrence (Table 2). In the multivariable analysis only, serum CRP, S100A12, BMI and systolic BP remained significantly associated (Table 3). The serum levels of CRP and S100A12 suggested that as these two markers increased, so did the probability of being a PTB patient (Odds Ratio (OR) 3.61, 95%CI 2.008–6.488, p = < 0.0001) and (OR 1.873, 95%CI 1.212–2.891, p = 0.004) respectively (Table 3). In summary, PTB patients had increased serum levels of CRP and S100A12 and both parameters are independent predictors of disease occurrence.

### Serum levels of CRP and S100A12 change longitudinally during DOTS treatment

**Figure 1:**
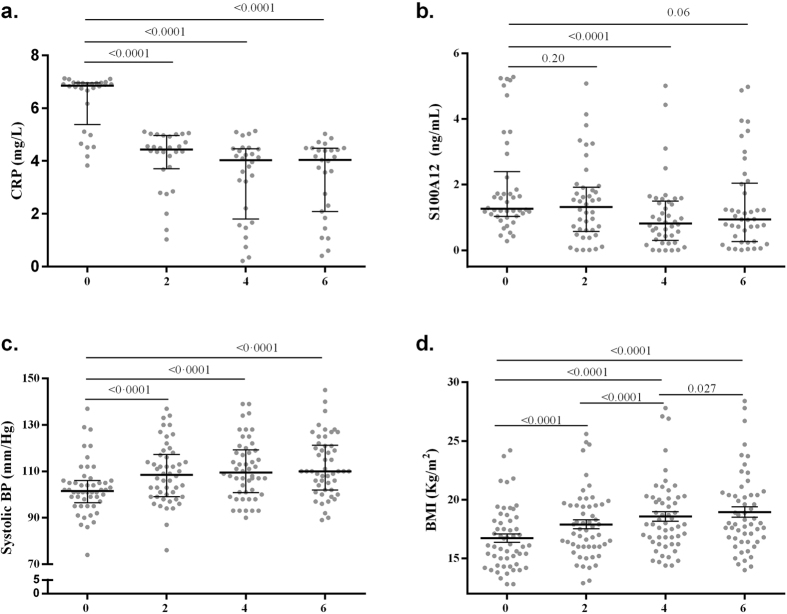
CRP, S100A12, systolic BP and BMI values change longitudinally during disease recovery under DOTS treatment. TB patients were followed up during the antibiotic treatment on average 6 months and the values of (**a**) CRP, (**b**) S100A12, (**c**) systolic BP and (**d**) BMI were compared over time at 0, 2, 4 and 6 months in those individuals for which the four measurements were available, significance was tested by Friedman and Wilcoxon signed rank tests in (**a–c**) and represented as the median ± interquartile range (IQR) or with repeated measure ANOVA with post hoc test in (**d**) and represented as mean ± standard deviation (SD). n = 28 in (**a**), 42 in (**b**), 50 in (**c**) and 55 in (**d**) individuals per time point. Time interval is given in months.

To further assess a causal relationship and to rule out confounding effects due to other unknown factors, we used a repeated measure analysis to determine whether serum CRP, and S100A12 change in conjunction with DOTS treatment at 0, 2, 4 and 6 months. As shown in (Fig. 1a) the serum levels of CRP significantly decreased after 2 months of DOTS treatment (4.40 ± 0.99 mg/L) compared to the levels at the time of diagnosis (6.75 ± 2.13 mg/L, p < 0.0001). While S100A12 significantly decreased after 4 months (0.822 ± 1.196 ng/mL) compared to the levels at the time of diagnosis (1.262 ± 1.358 ng/mL, p < 0.0001) (Fig. 1b). Similarly, significant changes were observed in systolic BP and BMI values (Fig. 1c,d).

### Patients with pulmonary TB showed higher neutrophil and lower lymphocyte counts; neutrophils were positive predictors of S100A12 serum levels

**Figure 2:**
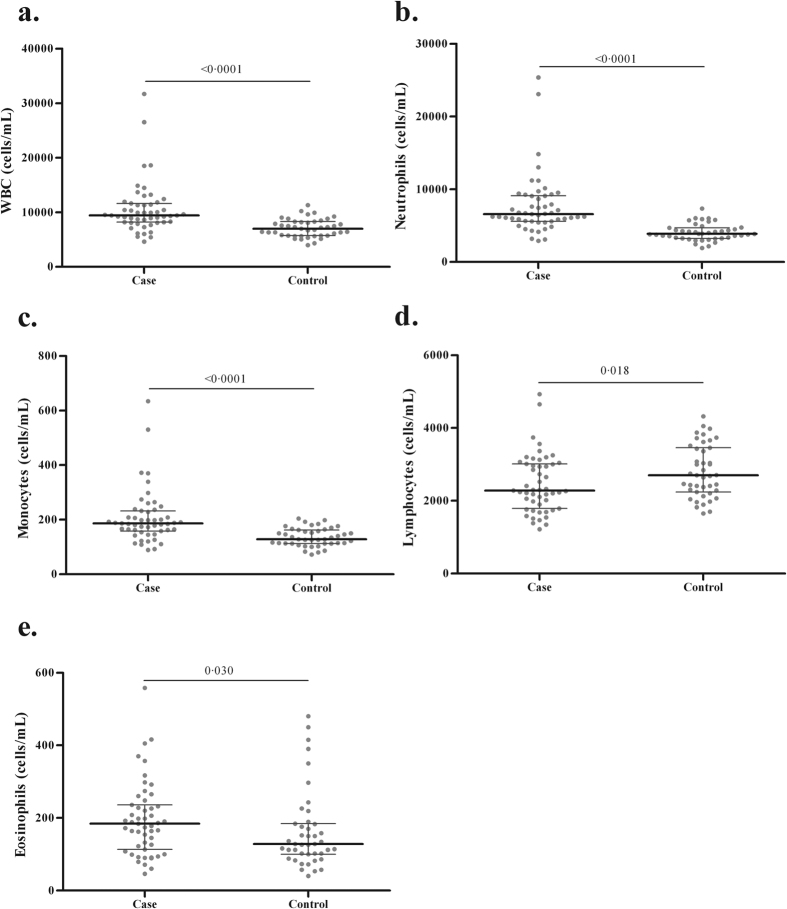
TB patients have increased neutrophil counts and decreased lymphocyte counts in their peripheral blood when compared to healthy controls. (**a**) Total WBC, (**b**) Neutrophil count, (**c**) Monocyte count, (**d**) Lymphocyte count and (**e**) Eosinophil count. Data are presented as the median ± interquartile range (IQR) and significance was tested with Mann Whitney U tests. n = 51 for cases and 43 for controls.

S100A12 is constitutively expressed by neutrophil granulocytes^11^ and several clinical studies have associated the serum levels of S100A12 with neutrophil activation and neutrophilic inflammation^30^,^31^. In order to evaluate whether the same pattern is present in PTB we measured the differential peripheral White Blood Cell (WBC) counts in a subgroup of TB cases and healthy controls that had this value available in their clinical records and compared first the differences in total cell numbers between the two groups and secondly we assessed the association of S100A12 in serum with the different cells populations by linear regression analysis. As shown in (Fig. 2a), a significantly higher WBC count was observed in patients when compared to healthy controls. This was mainly due to the increase of neutrophils and monocytes (Fig. 2b,c). In contrast, lymphocytes were decreased in PTB compared to controls (Fig. 2d). Next, we used univariable linear regression to show that neutrophil and eosinophil counts were positive predictors of S100A12 serum levels (β values 0.265 and 9.421, p = 0.016 and p = 0.012, Table 4 models 4 and 5, respectively). In contrast the associations with the lymphocyte and monocyte counts did not reach statistical significance. A subsequent multivariable analysis revealed that only the neutrophil count remained as significant positive predictor of the S100A12 serum levels (β value 0.265, p = 0.016) (Table 4, model 6).

### S100A12 is a positive predictor of the extent of alveolar infiltration in the CXR of patients with pulmonary TB

We recently developed an assessment scheme for grading disease severity in PTB by specifically considering the five CXR manifestations: lung involvement, alveolar infiltration, cavitation, lymphadenopathy and pleural effusion, these CXR features were differently associated with Body Mass Index (BMI) and sputum smear positivity, and changed to a different extent after 6 months of treatment^32^. We used this same assessment scheme to evaluate the association of these specific CXR features with CRP, S100A12, sRAGE and WBC using regression analysis. A univariable ordinal regression revealed that the monocyte count and serum S100A12 levels were positive predictors for the extent of alveolar infiltration (OR 1.01, 95%CI 1.00–1.01, p = 0.049) and (OR 1.37, 95%CI 1.10–1.71, p = 0.005 respectively). Blood neutrophil count was also a positive predictor, but on the borderline of statistical significance (OR 1.18, 95%CI 1.0–1.39, p = 0.050) (Table 5 models 1, 2 and 3, respectively). In contrast, only S100A12 levels were significantly associated with the extent of alveolar infiltration (OR 2.60, 95%CI 1.35–5.00, p = 0.004) in a multivariable model (Table 5, model 4). Thus, our data implicate that S100A12 independently predicts the extent of alveolar infiltration in the CXR of PTB patients. Furthermore, the probability of having infiltration in all four lung quadrants increases with rising S100A12 serum levels.

## Discussion

Recent studies suggest that neutrophils play a central role in the inflammatory response that causes PTB lung pathology^8^. In accordance, the results of our study demonstrate that PTB was associated with an increase in the serum concentration of S100A12, as well as with elevated blood neutrophil counts. Additionally, PTB was associated with a decrease in sRAGE serum levels. Importantly, although S100A12 along with CRP independently predicted PTB disease occurrence, only the serum concentration of S100A12 served as a predictor of alveolar lung infiltration evaluated by CXR.

The association of the serum S100A12 concentration with the extent of alveolar infiltration suggests that increased S100A12 serum levels reflect the neutrophilic lung damage seen in PTB patients. This is in accordance with recent reports describing that active PTB is characterized by an exudative reaction progressing to tuberculous pneumonia^33^. Also supporting this, serum levels of S100A12 and sRAGE are associated with other chronic pulmonary diseases such as chronic obstructive lung disease (COPD) and cystic fibrosis (CF), in which neutrophils are known to be considerably involved in the pulmonary inflammatory process and disease pathology^34^,^35^.

Considering the role of sRAGE in chronic inflammatory processes, decreased sRAGE serum levels were reported in COPD and rheumatoid arthritis patients^30^,^31^,^36^,^37^,^38^,^39^,^40^,^41^. Of note, in most chronic diseases decreased sRAGE is accompanied by an increase in S100A12 which is associated with disease severity^19^. Our results support the hypothesis that during active inflammation in PTB, neutrophil influx into the lung results in increased S100A12 concentrations. Further that serum S100A12 concentration might serve as an indicator to assess the extent of pulmonary inflammation. In longitudinal studies of lung injury, sRAGE and S100A12 serum levels returned to control levels after disease recovery^31^,^42^. Similarly in our study S100A12 serum levels returned to normal values during DOTS therapy. This normalization of S100A12 was accompanied by a concurrently increasing BMI, likely reflecting the improvement of the patient’s general condition during treatment^43^.

Recent evidence highlights the association of S100 proteins with neutrophil associated inflammation and their role as potential surrogate markers to assess lung inflammation and disease severity in PTB^44^. S100A8/A9 protein dimer is present in the inflammatory lung granulomas in active human PTB and in the TB mouse model these proteins mediate neutrophilic accumulation by inducing production of proinflammatory chemokines and cytokines^44^. Accordingly, our results not only suggest that neutrophils are the source of S100A12 but also that this protein is a predictor of alveolar infiltration, and we have recently shown that alveolar infiltration is associated with severity and infectivity in PTB^32^. Importantly, we measured CRP, a classical acute phase protein and indicator of immune system activity, although we found that CRP is a good predictor of disease occurrence, the serum concentration of this protein was not associated with any radiographic abnormality. These results are in line with a study that found no correlation between CRP and CXR abnormalities in PTB^45^. Thus, while CRP is produced in the liver and is a well-known marker of systemic inflammation^46^, S100A12 might provide information about lung inflammation that is different to systemic inflammation expressed by CRP. Alveolar infiltration might lead to scaring and long term pulmonary affection which could be preventable^47^. Accordingly, a recent systematic review found that TB is strongly associated with the presence of chronic respiratory disease in adults in TB endemic areas, highlighting the need for actions to improve long-term lung health in TB care and surrogate markers to assess lung involvement and damage^48^. Of note, we found no association between neutrophil counts and alveolar infiltration. During infection more than one-half of the neutrophils in the peripheral circulation are attached to the vascular endothelium^49^. As these cells migrate across the endothelium to inflammatory foci, they get activated; generate free radicals, and release granule contents and inflammatory mediators such as calgranulins^50^. Our data suggest that an association between neutrophil count and alveolar infiltration might exist but the results did not reach statistical significance and it could be explained by the small sample size because the neutrophil count was available only for 51 TB patients. However, although an association between neutrophil count and alveolar infiltration could not be ruled out, our results demonstrate that the association with S100A12 is stronger.

One limitation of our study is that although all the individuals used as controls were clinically healthy at the moment of sample collection, we are not certain about their status in terms of past exposure to *M. tuberculosis* or latency. Thus some might differ in their latency status what could eventually affect the results. In addition, we followed up the patients only for 6 months what does not allow assessment of any associations with long term outcome. In this study we reveal an association of S100A12 with lung involvement in PTB, however, similar associations have been shown with other inflammatory diseases^19^, thus, future studies should address the comparison of this immune marker across different pulmonary diseases.

Our findings suggest that the measurement of S100A12 serum concentration may help to stratify the extent of alveolar infiltration of PTB patients at the time of diagnosis, to identify cases that would benefit from strategies to avoid long-term lung impartment. This research has laid the ground work for further evaluate the potential application of serum S100A12 and S100 proteins for the assessment of lung involvement in patients with PTB

In conclusion our results highlight the role of neutrophils in the pathogenesis of smear positive PTB and demonstrate that S100A12 could serve as a novel target to assess the extent of alveolar infiltration in patients with PTB. Therefore the role of this molecule as biomarker for TB should be more accurately defined.

## Materials and Methods

### Study design, setting and participants

We carried out a cross sectional cohort and a self-controlled case series study in Mahavir hospital and Research Center in Hyderabad, India. For the purpose of calculating sample size, a pilot survey was performed with 25 TB patients and 15 healthy controls in March 2011. Our calculations indicated that, 94 and 45 individuals per group would be required for sRAGE and S100A12 respectively, to detect a significant difference according to the observed effect sizes and the influence of the potential confounders and effect modifiers with 80% power and 5% significance level. However more individuals were included to allow for missing data. The final recruitment period (July 26 2011- July 25 2013) resulted in a final sample size of 119 PTB patients and 163 healthy controls.

Criteria for inclusion as a case were: newly diagnosed pulmonary sputum smear positive TB disease and treatment naïve patients who entered the Hyderabad Directly Observed Treatment, Short-course (DOTS) program at Mahavir hospital. The diagnostic criterion for PTB was defined as the presence of one of the following: at least 2 initial sputum smear examinations positive for Acid-Fast Bacilli (AFB) or sputum examination positive for AFB and radiographic abnormalities consistent with active pulmonary TB^51^.

Criteria for inclusion as healthy control were: absence of apparent acute or chronic pulmonary diseases or diseases of other origin, and a negative history of TB disease. All healthy control individuals were from the same geographical origin, living in Hyderabad and were clinically in good health at the time of sample collection. Individuals with signs and symptoms suggesting active PTB or a history of prior anti-TB treatment were excluded from the study. All study participants gave written informed consent and the study was approved by the institutional ethics committee for bio-medical research at the Bhagwan Mahavir Medical Research Centre, Hyderabad, India. The methods used in this study were in accordance with the approved guidelines.

### End points, follow-up and variables

All PTB patients were prospectively followed up on average for 6 months from the beginning to the end of the treatment. The final end points were: death, loss to follow-up and end of therapy. The demographic data, including gender, age and BMI along with other relevant clinical data were recorded using the standard hospital’s DOTS program questionnaire. The main potential confounders and effect modifiers were: age, gender, BMI, blood glucose, smoking, alcohol consumption, HIV infection, blood pressure, lipid profile (cholesterol, Low Density Lipoprotein (LDL) and triglycerides), chronic kidney disease, cardiovascular disease and use of lipid lowering drugs which have been related to the risk of getting TB or to influence the levels of S100A12, sRAGE and esRAGE in serum^23^,^24^,^26^,^52^. The final treatment outcome was recorded as cured, died, failure or defaulted according to the India technical and operational guidelines for TB control^51^.

### Data sources and measurements

The demographic data were obtained directly from patients or their relatives and healthy individuals during an interview performed by a trained researcher at initial recruitment. Smoking and alcohol consumption were self-reported and assessed by including these questions into the standard DOTS questionnaire according to previously described^53^,^54^. Other relevant clinical data were obtained from the hospital’s TB control program records and medical staff. The blood pressure was measured with the MX2 digital automatic blood pressure monitor (Omron GmbH, Mannheim-Germany) and one peripheral blood sample was obtained after overnight fasting from healthy controls at initial recruitment and from TB patients at the start of therapy and after 2, 4 and 6 months. The serum was isolated and stored at −20 °C.

Complete peripheral blood count, lipid profile (total cholesterol, LDL cholesterol, triglycerides and HDL cholesterol) and creatinine were analysed by the hospital pathology service using Sysmex KX-21 blood analyser (Sysmex, Kone-Japan) and Konelab 20 clinical chemistry analyzer (Thermo scientific, Rockford, Illinois- USA) respectively. Fasting blood glucose was measured using the Accu-Chek Performa (Roche Diagnostics GmbH, Mannheim-Germany). Immune markers were determined with commercially available ELISA kits as follows: sRAGE (R&D systems, Minneapolis, Minnesota-USA), esRAGE (B-Bridge International, Tokyo-Japan), HMGB-1 (IBL international GmbH, Hamburg-Germany), S100A12/EN-RAGE (MBL international, Woburn, Massachusetts-USA), CRP (Thermo scientific), TNF-(α) and IFN-(γ) (BD biosciences, Heidelberg-Germany).

### Chest radiography

A CXR analysis was performed to every recruited patient at the time of diagnosis using a Siemens Heliophos D X-rays generator (Siemens, Mumbai-India). The CXR were evaluated as previously described^32^.

### Statistical methods

Normally distributed variables were expressed as mean ± SD, not normally distributed variables as median ± interquartile range, and statistical differences between two groups were analysed using a two-sided Student’s *t* test or a Mann Whitney U test. One way ANOVA with post hoc test or Kruskall Wallis and Mann Whitney U test with Bonferrini adjustment were used for normally and not normally distributed variables when more than two groups were compared. Binary logistic regression was used for adjustments for potential confounders in the cross sectional associations and for assessing predictors of disease occurrence. Univariable logistic regression models were done and those variables found to be predictors of disease occurrence were then analysed with hierarchical multivariable and backwards Likelihood Ratio (LR) regression models. Hosmer-Lemeshow goodness of fit test was used as goodness of fit for model fitting assessment and VIF values were used to assess multicollinearity. The best fitting models were chosen by analysing the change in the LR statistic after different variables were included or removed from the model. Cox & Snell and Nagelkerke R squares were reported to assess the proportion of variance explained by the predictors and different models. The longitudinal data were analyzed with repeated measures ANOVA with post hoc test or with Friedman test and Wilcoxon signed rank test for normally and not normally distributed variables respectively.

Linear regression was used to analyse the association of S100A12 with peripheral blood cell counts. In linear regression analysis ANOVA test was used as goodness of fit for model fitting assessment and the R^2^ test to assess the proportion of variance explained by the predictors. In linear and logistic regressions, deviance and partial residuals were used to identify potential outliers influencing the models. The association of alveolar infiltration and immune markers was assessed with univariable and multivariable ordinal regression analysis: the goodness of fit test was used for model fitting assessment, the amount of variation explained by the model with pseudo R^2^ and the proportional odds assumption was assessed with the test of parallel lines. SPSS 21 (SPSS Inc., Chicago, Illinois USA) was used for data analysis and values of *p* < 0.05 were considered significant. Graphics were done with GraphPad Prism Software version 5.00 for Windows (GraphPad Software, San Diego California, USA) and sample size and power calculations with OpenEpi version 3.01. The STROBE guideline was used to guide the report of this observational study.

## Additional Information

**How to cite this article**: Berrocal-Almanza, L. C. *et al*. S100A12 is up-regulated in pulmonary tuberculosis and predicts the extent of alveolar infiltration on chest radiography: an observational study. *Sci. Rep.* **6**, 31798; doi: 10.1038/srep31798 (2016).

## Figures and Tables

**Table 1:** Baseline characteristics, related parameters and serum levels of CRP, S100A12, sRAGE and HMGB-1 in TB cases and controls.

Variable	Case (n = 119)	Control (n = 163)	P value (Crude)	P value (Adjusted)
Age mean (SD), (Years)	26 ± 11	32 ± 10	<0.0001	—
Sex No. (%), male/female	58 (49)/61 (51)	82 (50)/81 (49)	0.81	—
BMI mean (SD), (Kg/m^2^)	16 ± 2.6	24 ± 4.5	<0.0001	<0.0001^a^
Smoking habits mean (SD), pack-year	0.21 ± 0.83	0.07 ± 0.28	0.043	0.004^a^
Drinking habits No. (%), (yes/no)	22 (19)/97 (81)	3 (2)/160 (98)	<0.0001	<0.0001^a^
Fasting blood glucose mean (SD), (mg/dL)	97 ± 30	104 ± 30	0.06	—
Blood pressure mean (SD), (mm/Hg)				
Systolic	103 ± 15	123 ± 17	<0.0001	<0.0001^a,b^
Diastolic	74 ± 10	80 ± 12	<0.0001	0.97^a,b^
Cholesterol mean (SD), (mg/dL)	167 ± 27	183 ± 33	<0.0001	0.73^b^
LDL mean (SD), (mg/dL)	104 ± 25	115 ± 25	<0.0001	0.77^b^
HDL mean (SD), (mg/dL)	41 ± 8.8	43 ± 33	0.37	—
Tryglicerides mean (SD), (mg/dL)	112 ± 38	136 ± 80	0.003	0.87^b^
Creatinine mean (SD), (mg/dL)	0.91 ± 0.13	0.96 ± 0.17	0.005	0.77 ^b^
CRP median (IQR), (mg/L)	6.74 ± 2.15	3.18 ± 3.23	<0.0001	<0.0001^a,b,c,d,e^
TNF-α mean (SD), (pg/mL)	13.4 ± 21.8	6.1 ± 20.4	0.004	0.02^a,b,c,d,e^
IFN-γ median (IQR), (pg/mL)	7.0 ± 18	3.7 ± 11	0.17	—
S100A12 mean (SD), (ng/mL)	2.332 ± 1.631	1.090 ± 1.050	<0.0001	<0.0001^a,b,c,d,e^
sRAGE mean (SD), (ng/mL)	0.74 ± 0.54	1.002 ± 0.563	<0.0001	0.003^a,b,c,d,e^
esRAGE mean (SD), (ng/mL)	0.06 ± 0.15	0.07 ± 0.10	0.65	—
HMGB-1 median (IQR), (ng/mL)	3.3 ± 5.5	3.0 ± 5.6	0.67	—

Adjusted for: ^a^Age, ^b^BMI, ^c^Smoking habits, ^d^Drinking habits and ^e^Systolic BP by binary logistic regression. (SD) standard deviation, (IQR) interquartile range.

**Table 2:** Univariable logistic regression analysis: predictors of disease occurrence.

Predictor	β	S.E	P value	Exp β (OR)	95%CI for Exp β
**Model 1** CRP	1.646	0.225	<0.0001	5.186	3.336–8.063
**Model 2** S100A12	0.681	0.108	<0.0001	1.974	1.592–2.443
**Model 3** sRAGE	−0.483	0.206	0.019	0.613	0.413–0.924
**Model 4** TNF-α	0.019	0.007	0.010	1.024	1.005–1.034
**Model 5** Age	−0.051	0.012	<0.0001	0.954	0.927–0.973
**Model 6** BMI	−0.650	0.073	<0.0001	0.523	0.452–0.603
**Model 7** Systolic BP	−0.081	0.011	<0.0001	0.924	0.903–0.944
**Model 8** Fasting blood glucose	−0.009	0.005	0.081	0.992	0.979–1.001
**Model 9** Smoking habits	−0.566	0.319	0.076	1.763	0.943–3.291
**Model 10** Drinking habits	2.487	0.629	<0.0001	12.02	3.505–41.23

Models parameters: Model 1 CRP: constant: −8.20, −2Log likelihood 185-Chi^2^ < 0.0001, Hosmer and lemeshow test <0.0001, R^2^ (Cox 0.49 and Nagelkerke 0.67). Model 2 S100A12: constant: −1.42, −2Log likelihood 328-Chi^2^ < 0.0001, Hosmer and lemeshow test 0.01, R^2^ (Cox 0.17 and Nagelkerke 0.23). Model 3 sRAGE: constant: 0.206, −2Log likelihood 405-Chi^2^ 0.016, Hosmer and lemeshow test 0.64, R^2^ (Cox 0.019 and Nagelkerke 0.026). Model 4 TNF-α: constant: −0.48, −2Log likelihood 375-Chi^2^ 0.016, Hosmer and lemeshow test 0.07, R^2^ (Cox 0.03 and Nagelkerke 0.04). Model 5 Age: constant: 1.17, −2Log likelihood 392-Chi^2^ 0.002, Hosmer and lemeshow test 0.82, R^2^ (Cox 0.06 and Nagelkerke 0.08). Model 6 BMI: constant: 12.05, −2Log likelihood 170.5-Chi^2^ < 0.0001, Hosmer and lemeshow test 0.88, R^2^ (Cox 0.55 and Nagelkerke 0.73). Model 7 Systolic BP: constant: 8.8, −2Log likelihood 312.1-Chi^2^ < 0.0001, Hosmer and lemeshow test 0.21, R^2^ (Cox 0.24 and Nagelkerke 0.33). Model 8 Fasting blood glucose: constant: 0.66, −2Log likelihood 403.8-Chi^2^ 0.048, Hosmer and lemeshow test 0.86, R^2^ (Cox 0.012 and Nagelkerke 0.017). Model 9 Smoking habits: constant: −0.302, −2Log likelihood 405-Chi^2^ 0.032, Hosmer and lemeshow test 0.001, R^2^ (Cox 0.01 and Nagelkerke 0.02). Model 10 Drinking habits: constant: −0.494, −2Log likelihood 358-Chi^2^ < 0.0001, Hosmer and lemeshow test 0.001, R^2^ (Cox 0.08 and Nagelkerke 0.11).

**Table 3:** Multivariable logistic regression analysis: S100A12 and sRAGE are independent predictors of disease occurrence.

Predictor	Β	S.E	P value	Exp β (OR)	95%CI for Exp β
CRP	1.289	0.299	<0.0001	3.61	2.008–6.488
S100A12	0.608	0.299	0.042	1.837	1.022–3.305
sRAGE	−1.668	0.910	0.067	0.189	0.032–1.122
TNF-α	0.012	0.017	0.473	1.012	0.980–1.046
BMI	−0.608	0.143	<0.0001	0.932	0.411–0.720
Systolic BP	−0.071	0.033	0.030	0.932	0.874–0.993

Model parameters: constant: 14.32, −2Log likelihood 42-Chi^2^ < 0.0001, Hosmer and lemeshow test 0.94, R^2^ (Cox 0.67 and Nagelkerke 0.92). Cases n = 110, Controls n = 160.

**Table 4:** Univariable and multivariable linear regression analysis: neutrophils are positive predictors of S100A12 serum levels.

Predictor	Β	S.E	P value	95%CI for β
**Model 1** WBC	0.240	0.078	0.011	0.068–0.412
**Model 2** Neutrophils	0.265	0.094	0.016	0.059–0.470
**Model 3** Monocytes	7.945	3.947	0.069	−0.742–16.63
**Model 4** Lymphocytes	0.975	0.474	0.064	−0.069–2.019
**Model 5** Eosinophils	9.421	4.126	0.012	2.541–16.32
Multivariable				
**Model 6** Neutrophils	0.265	0.094	0.016	0.059–0.470

Models parameters: Model 1: WBC. Constant 867, ANOVA 0.011, R^2^ 0.41. Model 2: Neutrophils. Constant 1417, ANOVA 0.016, R^2^ 0.36. Model 3: Monocytes. Constant 1596, ANOVA 0.069, R^2^ 0.20. Model 4: Lymphocytes. Constant 607, ANOVA 0.064, R^2^ 0.21. Model 5: Eosinophils. Constant 1614, ANOVA 0.012, R^2^ 0.40. Model 6: Constant 1417, ANOVA 0.016, R^2^ 0.36. Other variables in model 6: Lymphocytes, monocytes, eosinophils.

**Table 5:** Univariable and multivariable ordinal regression: S100A12 is a positive predictor for the extent of alveolar infiltration in the CXR of patients with pulmonary TB.

Predictor	Β	S.E	P value	Exp. β (OR)	95% for Exp. β (OR)
**Model 1** Neutrophils	0.165	0.084	0.050	1.18	1.00–1.39
**Model 2** Monocytes	0.007	0.003	0.049	1.01	1.00–1.01
**Model 3** S100A12	0.317	0.112	0.005	1.37	1.10–1.71
Multivariable					
**Model 4** Neutrophils	−0.106	0.228	0.643	0.90	0.58–1.41
Monocytes	0.011	0.010	0.272	1.01	0.99–1.03
S100A12	0.956	0.333	0.004	2.60	1.35–5.00

Dependent ordinal variable alveolar infiltration (None, 1 quadrant, 2 quadrants, 3 quadrants and 4 quadrants). Models parameters: Model 1 −2Log 128 Chi^2^ 0.052, Goodness of fit 0.47, Pseudo R^2^ (Cox and snell 0.075, Nagelkerke 0.081), test of parallel lines 0.516; n = 51. Model 2 −2Log 118 Chi^2^ 0.051, Goodness of fit 0.66, Pseudo R^2^ (Cox and snell 0.076, Nagelkerke 0.081), test of parallel lines 0.490; n = 51 Model 3 −2Log 295 Chi^2^ 0.005, Goodness of fit 0.79, Pseudo R^2^ (Cox and snell 0.068, Nagelkerke 0.073), test of parallel lines 0.089; n = 112. Model 4 −2Log 70 Chi^2^ 0.002, Goodness of fit 0.83, Pseudo R^2^ (Cox and snell 0.39, Nagelkerke 0.42), test of parallel lines 0.47; n = 51. Reference category: alveolar infiltration 4 quadrants.
